# Forty-Year Biomonitoring of Environmental Contaminants in Russian Arctic: Progress, Gaps and Perspectives

**DOI:** 10.3390/ijerph191911951

**Published:** 2022-09-21

**Authors:** Alexey A. Dudarev, Jon Oeyvind Odland

**Affiliations:** 1 Northwest Public Health Research Center, 191036 St. Petersburg, Russia; 2 Department of Public Health and Nursing, NTNU, The Norwegian University of Science and Technology, 7034 Trondheim, Norway; 3 Department of General Hygiene, I.M. Sechenov First Moscow State Medical University, Ministry of Health of Russia, 119992 Moscow, Russia; 4 Institute of Ecology, National Research University Higher School of Economics, 101000 Moscow, Russia

**Keywords:** biomonitoring, Russian Arctic, biota, wildlife species, traditional foods, indigenous people, human tissues, blood, breastmilk, contaminants, POPs, DDTs, PCBs, metals, exposure, health risks

## Abstract

This article presents a comprehensive survey of the studies on the biomonitoring of persistent organic pollutants (POPs) and metals in biota and human tissues on the territory of the Russian Arctic. It is concluded that a relatively small number of studies were carried out during the last 40 years; for several Russian Arctic regions there is no data up to date, and for some regions the data are scarce, with most of the studies carried out in 1990s, followed by the large-scale GEF/AMAP/RAIPON project of 2001–2004 covering four regions. After that, single projects have been implemented in a few regions. Only the Nenets okrug and the Chukotka okrug (and hardly the Murmansk oblast) can be attributed as the regions where the biomonitoring of contaminants was carried out during last decades on several occasions, and for which the content of POPs and metals in biota and the human organism was assessed in 12–15-year dynamic trends (at least “at three points”). For the rest of the Russian Arctic territories, only fragmentary “cross-sections” of biomonitoring data is available, mainly obtained in the 1990s or early 2000s, which do not allow judging either the dynamics of the processes or the current state of affairs. The overwhelming majority of the studies in the Russian Arctic (more than 90%) were carried out within the framework of international projects, i.e., with cofinancing, assistance and contribution (including laboratory analyses) from the foreign colleagues and partners. The shortcomings of the Russian system of biomonitoring, including the weakness of the laboratory and research base, are considered. Perspectives of the Russian Arctic biomonitoring are discussed in detail, with the proposal of the elaboration of the national Russian Arctic Contaminants Program (RACP).

## 1. Introduction

### 1.1. Biomonitoring of Persistent Toxic Substances (PTS)

Within the scope of long-term international scientific collaboration in the circumpolar Arctic, the biomonitoring of environmental contaminants is understood as a systematic measurement of persistent toxic substances (PTS) in human bodily fluids and tissues, as well as in biota (wildlife species). Biomonitoring is the basis for assessing human exposure to contaminants, as well as for studying the biological effects and health risks associated with the exposure to PTS accumulated in the human organism.

Regarding human biological media, this review focuses on the levels of contaminants in blood (and breastmilk) because, in the context of circumpolar collaboration, all participating countries have collected blood samples (and less frequently breastmilk) for international comparison. Hair, nails, urine, feces and other matrices are not discussed here.

Along with human biological media, the objects of the biomonitoring of contaminants in the Arctic include various wildlife species used by the Arctic peoples as food products. Local species of flora and fauna, being included in food chains (terrestrial, freshwater or marine), are exposed to PTS through bioaccumulation and biomagnification processes. Consumption of local foods which contain elevated concentrations of contaminants leads to the increased exposure and body burdens of the population to these contaminants.

The biomonitoring of contaminants in the Arctic covers two main groups of PTS: persistent organic pollutants (POPs) and metals. POPs are represented mainly by polyhalogenated hydrocarbons, some of which are included in the list of the Stockholm Convention as the most dangerous compounds banned for production, use and transportation, and which are subjected to extermination. All POPs are xenobiotics, but not all of them are products of intentional chemical synthesis, due to which the pesticides and technical additives were produced and effectively used in agriculture and industry for decades all over the world, circulated and accumulated in the planet’s environment, and gradually increasing the impact and danger to the living beings and humans. Partially, POPs have entered and continue to enter the planet’s biosphere as byproducts of various industrial productions, with emissions from power plants and boilers running on fossil fuels, with waste incineration, fires, etc. Some banned POPs are still produced in a number of countries and continue to be used, in particular in agriculture and for the control of malaria and other vector-borne diseases.

Metals (including mercury, lead and cadmium) naturally occur widely in nature, and exist in rocks, soil, water and biota. Metals are transported over vast distances due to rock weathering, forest fires and volcanic eruptions. Industrial sources (e.g., mining, ferrous and nonferrous metallurgy, fuel and waste combustion) account for one to two thirds of the total metal contamination of the Arctic.

PTS, represented by substances and compounds of an inorganic or organic nature, natural origin or xenobiotics, are characterized by a combination of several specific features; they are:

- Stable in the environment (for years or decades), resistant to thermolysis, photolysis, hydrolysis and chemical and bacteriological degradation;
- Could be transferred globally (thousands of kilometers); long-range transport of PTS to the Arctic occurs with atmospheric flows, rivers, seas and ocean currents;
- Able to bioaccumulate, reaching high levels in the upper links of food chains due to biomagnification processes; concentrations of PTS in the organisms of top predators can be from thousands to millions times higher than those in the air, soil, water and organisms of the lower tiers of the food chains;
- Extremely slowly metabolized and excreted from organisms, which contributes to the long-term accumulation of significant doses of PTS, even at their low concentrations in food. This phenomenon has gained particular importance in assessing the exposure to PTS of the indigenous people of the Arctic, whose diet is mainly based on the consumption of local (country, traditional) foods obtained by hunting and fishing;
- Toxic and hazardous to health in low (and ultra-low) concentrations. The lipophilicity of many POPs contributes to their accumulation in adipose tissue and lipid-rich organs (including the brain and spinal cord). The high permeability of the blood–brain barrier for many POPs predetermines the risk of their direct toxic effect on the central nervous system and the endocrine sphere. An increased content of POPs in the body can provoke serious disorders in the neuroendocrine, immune and reproductive spheres, in the processes of fetal development and in antitumor resistance. Some POPs are “hormonal mimics” that inhibit the production of natural hormones and disrupt the normal processes regulated by the endocrine glands. Many POPs penetrate the placental barrier, entering the child’s body during fetal development and in infancy—with mother’s milk.

### 1.2. Circumpolar Biomonitoring of Contaminants: Historical Milestones

The biomonitoring of contaminants in the circumpolar Arctic began in 1960 with the first study in Alaska. The DDT and DDE levels were analyzed in the local foods and human body fat in Eskimos, who lived in isolated small settlements where there was little or no use of insecticides and who ate food of local origin with maximal intake of animal fat. The food samples were collected in the villages of Shungnak, Kotzebue, Gambell, Hooper Bay and Point Hope. The studied foods which made up the major portion of the Eskimos’ diet included various fresh and dried fish, eider duck, white owl; fat, oil or meat from beaver, caribou, moose, seal, bearded seal, walrus, whale, beluga and polar bear; and miscellaneous foods including cranberries, salmonberries and wild rhubarb [1].

Starting from the 1960s, the first studies on POPs in birds, marine mammals and fish were carried out in some regions of the circumpolar Arctic. During the Alaskan expedition in summer 1966 along the Yukon River (from Circle, Alaska to Castle Rock, Y.T.), the samples of eggs, fat, muscle, liver, brain of Alaskan Peregrines and the prey of peregrines (boreal birds, seed-eating and insectivorous passerines, sandpipers) were collected and analyzed (by electron gas chromatography) for DDTs and dieldrin. The authors concluded that their data demonstrated that, even in so remote a region as interior Alaska, the peregrine’s food chain is contaminated with significant, measurable quantities of persistent residues of the chlorinated hydrocarbons [2]. From 1967 to 1970, the data on Falco peregrinus eggs were collected from three geographic Alaskan populations: on Amchitka Island in the Aleutians, along the Yukon River in the interior taiga and along the Colville River in the foothill tundra of the Arctic Slope. It was concluded that changes in eggshell thickness and the pesticide residues reflected different degrees of exposure to DDT. There was a significant negative correlation between shell thickness and DDE content in peregrine eggs [3].

The first data on POPs in marine mammals were published by Holden in the *Nature* journal in 1967 [4], which demonstrated significant levels of DDT and dieldrin in the blubber of seals and porpoises in the Scottish and Canadian North Atlantic. Soon, the detailed report appeared on worldwide seals contamination by POPs. Seal blubber analyses have shown that Arctic, Antarctic and Pacific Ocean waters had a lower concentration of organochlorine pesticide residues than did the waters of the Baltic Sea, North Sea and Irish Sea; dieldrin and PCB levels were the highest around Great Britain; the highest DDT levels were recorded in the Baltic and the Gulf of the St. Lawrence [5]. The detailed reports on DDT-related compounds and PCBs in the ringed seal and beluga from Canada and Greenland were published in the early 1970s [6,7,8]. Also in the early 1970s, the first publications on elevated levels of POPs in northern freshwater and anadromous fish species appeared [9,10]; the latter publication was the first to report OCs in polar bears.

The biological effects of organochlorine compounds in Arctic ecosystems have not previously been studied, with the exception of the impact on birds of prey, the decline in populations of which in the Arctic and temperate zones of the Northern Hemisphere, due to the beginning of the widespread use of pesticides, has been documented since the mid-1960s. Concerns about the potential human health impacts of POPs in the Arctic first appeared in the 1980s, when it was discovered that the breastmilk of indigenous northern Quebec (Inuit) women contained extremely high PCB levels (3–5 times higher than that of white women of southern Quebec) [11]. The results of studies of POPs in the diet of the Inuit of northern Canada in the late 1980s and early 1990s [12] demonstrated that namely the dietary route of human exposure to POPs determines the high levels of contaminants in the breastmilk and blood of indigenous Arctic peoples. The revealed patterns caused the need for additional information about contaminants in “traditional food products” and gave a powerful stimulus for further research in the circumpolar Arctic, including the beginning of such research in Russia.

### 1.3. Establishment of AMAP and Human Health Assessment Group

At the meeting in Rovaniemi (Finland) in June 1991, the ministers of eight Arctic countries (Canada, Denmark, Finland, Iceland, Norway, Russia, Sweden and the USA) signed the Declaration on the Protection of the Arctic Environment. Moreover, the Arctic Environmental Protection Strategy (AEPS) was adopted at this meeting, which stated that “The Arctic is highly vulnerable to contaminants, and most of its people and culture are directly dependent on the health of the region’s ecosystem.” As part of the Declaration, the Arctic Monitoring and Assessment (AMAP) was established to monitor contaminants and assess their impact on all components of the Arctic environment.

Under the umbrella of the AMAP, in October 1992, the Human Health Assessment Group (HHAG) was formed in Nuuk (Greenland), which identified POPs and metals as priority contaminants subjected to biomonitoring in the Arctic. For almost 30 years, the AMAP HHAG has been the “headquarters for coordination” of international circumpolar biomonitoring of contaminants; it generalizes and systematizes the results of the national monitoring studies of POPs and metals in human media and biota food webs, examines the peculiarities of PTS bioaccumulation and biomagnifications in local food chains (terrestrial, freshwater, marine), assesses the intake of PTS by the humans (exposure levels) and evaluates the effects of exposure to contaminants and human health risks at various territories of the circumpolar Arctic.

The AMAP HHAG has prepared and published five reports of “Human Health in the Arctic”: 1998, 2002, 2009, 2015 and 2021 [13,14,15,16,17]; the AMAP 2015 report was translated into Russian and published in 2018 [18]. The chronology of the AMAP reports up to 2015 are inclusive, and their quintessence was described in the Russian journal of *Human Ecology* [19].

### 1.4. Geography of Biomonitoring of Contaminants in the Russian Arctic

The Russian Arctic territory (called by the government as the “Arctic Zone of the Russian Federation”) today, in 2022, consists of 11 separate territories: Murmansk Oblast, Karelia republic (northern districts), Arkhangelsk Oblast (northern districts), Nenets autonomous okrug, Komi republic (northern districts), Yamalo-Nenets autonomous okrug, Taimyr Dolgano-Nenets District of the Krasnoyarsk kraj, Turukhansky District of the Krasnoyarsk kraj, Evenkiysky District of the Krasnoyarsk kraj (northern areas), Yakutia (Sakha) republic (northern districts) and the Chukotka autonomous okrug. 

**Figure 1:**
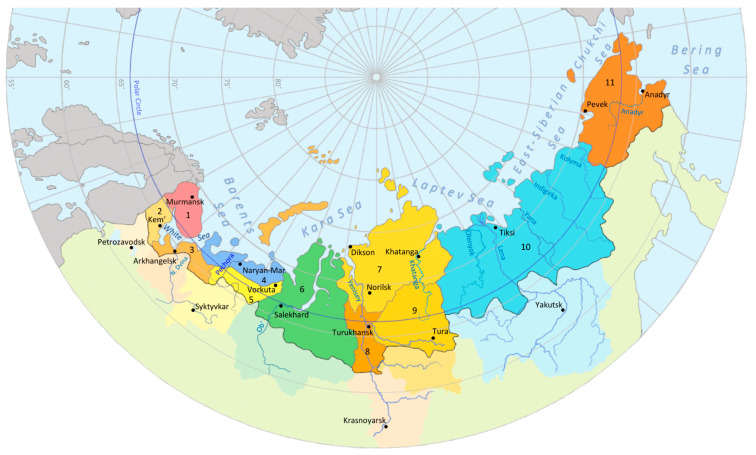
Map of the Russian Arctic territory (“Arctic Zone of the Russian Federation”): 1—Murmansk Oblast; 2—Karelia republic (northern districts); 3—Arkhangelsk Oblast (northern districts); 4—Nenets autonomous okrug; 5—Komi republic (northern districts); 6—Yamalo-Nenets autonomous okrug; 7—Taimyr Dolgano-Nenets District of the Krasnoyarsk kraj; 8—Turukhansky District of the Krasnoyarsk kraj; 9—Evenkiysky District of the Krasnoyarsk kraj (northern areas); 10—Yakutia (Sakha) republic (northern districts); 11—Chukotka autonomous okrug. (*The map was designed by Alexey V. Dozhdikov, research assistant from the Northwest Public Health Research Center, St-Petersburg, Russia*).

Generally, the territory of the Russian Arctic is located above the latitude 64° N (Figure 1). In this review we do not consider data obtained from regions (or districts) located to the south of the designated territories. Below, we present a spatiotemporal “picture” of all the studies (which we managed to find) carried out in the Russian Arctic (since the beginning of 1977 and up to 2021) related to the biomonitoring of contaminants both in biota and in the human body. The summarized information is also presented in Table 1 and Table 2.

## 2. Beginning of the Sea Fish and Seabirds Biomonitoring in the Russian Arctic

The biomonitoring of contaminants in the territory of the Russian Arctic started in Murmansk oblast in 1977, when, in the southern part of the Barents Sea near the northern coast of the Kola Peninsula, employees of the Murmansk Institute of Marine Biology (Tatiana Savinova and colleagues) for the first time analyzed the samples of several species of sea fish and invertebrates for DDT contamination. The results of these studies were first published in 1981 as a short article in the *Russian Journal of Hydrobiology* [20]. In 1979 and 1983, samples of various types of marine fish and birds were collected at several spots on the northern coast of the Kola Peninsula; the results of the analysis for DDTs and PCBs (in comparison with the circumpolar regions) were published in 1990 in the form of a scientific report by the Kola Science Center of the USSR Academy of Sciences, “Chemical pollution of the northern seas”. In 1991, the report was translated into English and published by the Canadian Institute of Scientific and Technical Information in Ottawa [21].

Norway and the Soviet Union signed The Agreement on Environmental Cooperation in 1988, and later signed a similar agreement between Norway and Russia in 1992. The Joint Norwegian–Russian Commission on Environmental Cooperation was established, with annual meetings. Working groups were initiated to increase collaboration on different environmental topics, e.g., air pollution, marine environment and radioactive pollution. This collaboration contributed to the harmonization and development of scientific methodology and databases [22].

The beginning of Norwegian–Russian seabird expert group cooperation was marked by the preparation and publication of the joint report, “Chemical pollution in the Arctic and sub-Arctic marine ecosystems: an overview of current knowledge” with Tatyana Savinova as the lead author [22]. The report was focused on the data obtained by that time (literature review and own results) concerning POP and metal levels in circumpolar Arctic biota, including Russian data on seabirds and sea fish sampled in the period 1977–1989 in several spots on the northern coast of the Kola peninsula (southern part of the Barents Sea).

As part of the Norwegian–Russian project “Environmental Contaminants in Arctic Seabirds” (with participation of Tatyana Savinova and Vladimir Savinov from the Murmansk Marine Biological Institute), which was launched in 1991 with the aim of providing reliable mapping of contamination levels in seabirds, two international expeditions to seabird colonies in the Barents Sea were carried out in 1991 and 1992. The expedition of 1991 was aimed at the analysis of legacy POPs in bird samples and covered Norwegian islands and part of Norwegian northern mainland coastline, but also included the Russian Franz-Josef Land. The results of the study were published in 1995 [36].

During the other expedition of 1992 to various seabird colonies in the Barents Sea, thirteen species of seabirds belonging to five families were collected on the Norwegian territory and in several Russian spots in the Barents Sea: Franz-Josef Land, the southern extremity of Novaya Zemlya, the Nenets okrug islands (Vaygach, Dolgy, Kolguev) and the northern coast of the Kola peninsula. The aim of this study was the analysis of six metals (Cd, Zn, Cu, As, Se and Hg) in the tissues of seabirds, and the results were published in 2003 [23].

Several species of seabirds were caught on islands of the Seven Islands Archipelago (northern coast of Kola peninsula) in the summer of 1992 and 2002. Hepatic tissue samples collected in 1992 were analyzed for POPs at the Institute of Pharmacology and Toxicology, Norwegian Veterinary College (Oslo, Norway); samples collected in 2002 were analyzed for POPs at the Centre of Environmental Chemistry “Typhoon” (Obninsk, Russia). During the decade period, a statistically significant decrease in the residue levels of the analyzed POPs was found in a majority of the sampled birds [26].

The Japan–Russian project (Ehime University, Matsuyama, Japan and the Institute of Biological Problems of the North, Russian Academy of Science, Magadan, Russia) was carried out in Chaunsky district of coastal Chukotka, where seabirds belonging to three orders, four families and eleven species were obtained during June–July 1993 to determine concentrations of four essential metals (Fe, Mn, Zn and Cu) and two toxic metals (Cd and Hg) in the selected bird tissues; the results were published in 1996 [48].

In June 1994, the Russian–German expedition on the research vessel “Dalniye Zelentsy” collected samples of 15 marine fish species in the southern part of the Barents Sea (Rybach’ya bank near the northern coast of Kola peninsula). Six metals (Cd, Pb, Hg, Ni, Cu and Zn) in fish tissues were analyzed in the University of Oldenburg laboratory (Germany); the results were published in 1999 [27] including three orders, four families and eleven species of fish.

## 3. Beginning of Inland Biota Biomonitoring in the Russian Arctic

In the 1970s, David Peakall discovered that OCs could be extracted from the lipids of the membranes of dried bird eggshells. This made it possible to relate eggshell thinning to pesticide levels, even when the egg contents were not available [85]. Specimens were collected from museums and private collectors globally. In 1979, a large dataset of eggshell index and DDE levels in egg membranes were reported, including data on eggs (from the Moscow Zoological Museum) of *Falco peregrinus leukogenus* from the Russian Arctic, namely from the mouth of the Indigirka river (Yakutia; 71° N, 146° E) collected annually in 1961–1965, and from the Pyasina river (Taimyr; 73° N, 91° E) collected in 1966. The Yakutian DDE levels in the eggshells were in the range of 80–180 ppm (lipid), while the Taimyr levels were 180–200 ppm (lipid) [85].

Studies on a population of peregrine falcons on the Kola Peninsula have been ongoing since 1977. Within the US–Russian collaboration, an international expedition in 1991 along the upper and middle course of Ponoy river (eastern part of Kola peninsula, Murmansk oblast) was organized and aimed at visiting most of peregrine eyrie sites during the egg stage and “sampling” the eggs. The eggs were brought to the US for analysis of DDTs, PCBs, PCDD/Fs, HCHs, HCB, toxaphenes, chlordanes, dieldrin and mirex. The eggs revealed relatively low concentrations of DDE (geomean 3.5 mcg/g) and of other OCPs. The DDE levels and eggshell thinning (11.4%) were similar to those reported in the peregrine eggs from Alaska. Levels of PCBs were higher than DDE concentrations, comparable to Fennoscandian falcons, but higher than those found in Alaska (3–21 mcg/g ww; geomean of 7.3 mcg/g ww). Concentrations of PCDD/Fs and planar PCBs in eggs were also relatively high, with combined TEQ levels of 86–640 pg/g ww [24].

In the frame of the Norwegian–Russian collaboration (University of Tromso with two Institutes of the Russian Academy of Sciences: Institute of Problems of Industrial Ecology of the North, Apatity, Murmansk oblast, and the Severtzov Institute of Ecology and Animal Morphology, Moscow, Russia), the studies of 1991 and 1992 on the fish of three lakes (Kuetsjarvi, Bjarnevatn and Vaggatem) in a watercourse of the Pasvik River system in the border region between Norway and Russia, in the vicinity of the Nickel town mining and metallurgic activity, were organized. The metal content from the muscle and liver were studied in different fish tribes at the laboratory of the Norwegian Institute for Nature Research. Concentrations of Cd and Ni in fish tissue increased with increasing proximity to the smelters, whereas the other elements showed similar concentrations at the three localities. The levels were within the ranges reported for other areas, but higher than unpolluted study sites [25]. 

During investigations of the Lower Lena River region in August–September 1992 by the Institute of Global Climate and Ecology of the Russian Federal Service for Hydrometeorology and Natural Environmental Monitoring (Russian Academy of Sciences, Moscow, Russia), the levels of background pollution of the atmosphere, soil, vegetation and biota were studied. The levels of DDT, DDE, α-HCH and γ-HCH were analyzed in two samples of ptarmigan (*Lagopus lagopus*) and four samples of freshwater muksun (*Coregonus muksun*) [43]. 

Within the US–Russian project (with participation of the Extreme North Research Institute, Norilsk, Russia) during the summer of 1993, 50 samples of freshwater fish (char, whitefish and burbot) were collected from four lakes located at southern foothills of the Byrranga mountains in the northern part of Taimyr peninsula, along with samples of lemmings taken at two sites, and soils, sediments and vegetation taken at seven sites. All samples were stored frozen and then shipped by air to the Battelle Marine Sciences Laboratory in the US, where the fish samples were analyzed for eight metals (As, Hg, Pb, Cd, Ni, Cu, Se and Zn) [41].

In 1991, the “Regional Center Monitoring of the Arctic” (RCMA) was established in St-Petersburg, Russia, on the basis of the Arctic and Antarctic Research Institute of the USSR State Committee for Hydrometeorology (in 2006, the RCMA was reassigned to the North-Western Branch of the SPA “Typhoon”). In the early 1990s, the RCMA was the key organizer and implementer of the first large-scale assessment of the contamination of the Russian Arctic seas coastline; this investigation was a part of the Russian AMAP National Implementation Plan. During the period of 1994–1995, samples of vegetation and biota were collected by the RCMA in many spots along the whole of the Russian Arctic coastline (of the Barents, Kara, Laptev, East Siberian and Chukchi seas), mainly at the deltas of large rivers and on the nearby islands. The results of the chemical analysis of the POPs and metals (in RCMA laboratory) in the sampled biota (terrestrial and seabirds, fish and mammals, including reindeer) were published as the reports of the RCMA and Rosgydromet in 1994–1996 [86,87,88,89,90] and were included in the first AMAP-1998 report.

The RCMA provided levels for Cd, Pb and Hg in the muscle and liver tissues of birds across Arctic Siberia (done during 1995). In the muscle tissue, concentrations of all metals decreased, with highest levels in the carnivores, but liver tissues provided no differences. Some geographical differences were observed, with higher Pb levels in the east. The same trend was observed for Cd in the muscle and liver of omnivores, and for Cd and Pb in the liver tissues. Moreover, the data on Cd, Pb and Hg in several fish species (sea, anadromous and freshwater) from various locations (sampling 1994–1995), and in liver, kidney and muscle of reindeer (*Rangifer tarandus*), were presented. Concentrations of Pb in the muscle and liver of reindeer were generally higher in the eastern part of the Russian Arctic; there was no similar trend for Cd [13].

PCBs, DDTs, HCHs, CBs and CHLs were analyzed by the RCMA in the tissues of reindeer, waterfowl and other terrestrial birds. The Russian data are sparse due to small numbers. An interesting observation was the difference between two years (1994 and 1995), with only one year difference, thus making it difficult to compare with other countries. However, the 1994 results were generally higher than for Canadian caribou and Svalbard reindeer. It should be noted that the PCBs in the Russian reindeer were based on only seven congeners (PCBs 28, 52, 101, 118, 153, 138 and 180), which were major congeners in Canadian caribou fat [13].

## 4. Beginning of Marine Mammals Biomonitoring in the Russian Arctic

Studies on the POP contamination of marine mammals in three Russian Arctic seas (Barents Sea, White Sea and Kara Sea) began almost simultaneously in the early 1990s.

Blubber samples from a few dozen of the Eastern harp seal (*Phoca groenlandica*) were obtained during the Norwegian expedition in April and May 1993 in the southern Barents Sea (East Ice) to the north of Cape Kanin (Nenets okrug). Significantly higher concentrations of the three major pollutants (∑PCB, ∑DDT and ∑CHL) were found in adults as compared to the juveniles, while no such relations were found for ∑HCH or HCB [37].

In the frame of the Finnish–Russian project, ringed seals (*Phoca hispida*) were sampled in the winter of 1993 in Sorokskaya Guba (near Belomorsk city in northern Karelia) of Onega Bay of the White Sea, and also in Lake Ladoga (Russia) and Lake Saimaa (Finland). The blubber samples were taken from seals that had died naturally or drowned in fishing gear. DDTs, HCHs, HCB, CHLs and PCBs were analyzed in the samples. It was concluded that, of the three areas studied, the highest concentrations of PCBs and DDTs in ringed seal blubber were found in Lake Saimaa; Lake Ladoga samples were contaminated moderately; the Arctic White Sea ringed seals indicated the lowest pollution due to long-range transport [33].

Under a cooperative Japan–Russian project in April 1995, 38 ringed seals were collected in Taimyr near Dikson Island (Kara Sea). The collected samples of blubber were analyzed for POPs. The levels of DDTs and PCBs were significantly higher than comparable results from the Canadian and Norwegian Arctic. It was predicted that significant local sources were present. Concentrations of CHLs and HCHs in ringed seals were similar, probably because of the global transportation to the Arctic region [42].

Within the Canadian–Russian project, the collection of tissues of several species of seals was conducted in three areas of the White Sea Basin during the 1998 and 2001 expeditions by the Northern Department of the Polar Research Institute of Marine Fishery and Oceanography (Arkhangelsk). In 1998, about 50 samples of harp seals, ringed seals and bearded seals were collected in the White Sea: ringed and bearded seals in the outer Dvina Bay (Arkhangelsk oblast) and harp seals near the southern extremity of the Kola Peninsula (Murmansk oblast). In autumn 2001, 23 ringed seals were collected in northwest part of the Onega Bay (Kemsky district of northern Karelia). In addition, blubber samples from 10 harp seal pups collected near the southern extremity of the Kola Peninsula (Murmansk oblast) in March 1992 were included to examine the temporal trends of OCs [28].

Moreover, several marine fish prey species (navaga, herring, bullrout, smelt and cod), as well as invertebrates, were collected in 1999–2000 from the research vessel “Professor Vladimir Kuznetsov” (Zoological Institute, St-Petersburg) in Chupa Guba of Kandalaksha Bay (Loukhsky district of northern Karelia) and the outer Dvina Bay (Arkhangelsk oblast) of the White Sea. The collected samples were analyzed for POPs in the “Typhoon” laboratory (Obninsk, Russia). It was shown that ∑PCB and p,p-DDE concentrations in ringed seals from eastern Svalbard and the southern Barents Sea were 2–3 times higher than in this study. PCB and ∑DDT concentrations in blubber were similar to levels from Svalbard and East Greenland. All major OCs, except HCH isomers, were higher compared to previous results in the Canadian and Alaskan Arctic, but lower than studies from the Kara Sea. A reduction of approximately 33% for ∑DDT to 60% for ∑PCB in the blubber of harp seal pups collected in 1992 compared with 1998 was expected, as declining concentrations ∑DDT in seawater were reported in inflowing rivers to the White Sea in the 1980s and early 1990s [28]. 

Under the auspices of the Norwegian–Canadian–Russian collaboration, samples of the blubber of adult ringed seals from the four areas of the Russian Arctic (White Sea, Barents Sea, Kara Sea and Chukchi Sea) were collected in 2001–2005 and analyzed for POPs at the Centre of Environmental Chemistry, S.P.A. Typhoon (Obninsk, Russia). The highest concentrations were found in the Kara Sea compared with those found in the other three areas of the Russian Arctic, while the highest mean concentrations of ΣHCHs and PCDD/Fs were found in the blubber of ringed seals from the Chukchi Sea and the White Sea, respectively. No significant differences between ΣPBDE concentrations were found in the blubber of ringed seals from the three study areas of the European part of the Russian Arctic, but the PBDE contamination level in ringed seals from the Chukchi Sea was 30–50 times lower [34].

## 5. Beginning of Human Biomonitoring in the Russian Arctic

The first data on the content of POPs and metals in human biological media were obtained in 1993–1996, also within the framework of the Russian–Norwegian cooperation under the auspices of AMAP, with participation and the active promotion of Jon Oyvind Odland from Tromso University. In the period 1996–1999, the first publications appeared on the results of these studies: on the content of essential trace elements and toxic metals in maternal and cord blood sampled in the Murmansk Oblast and Arkhangelsk Oblast [58,59,60,61,91], on the content of POPs in the breastmilk of mothers from the cities of Murmansk and Monchegorsk [57], Arkhangelsk, Severodvinsk (Arkhangelsk oblast) and Naryan-Mar (Nenets okrug) [70] (data on 5–7 year dynamics of POPs content in breastmilk from the Murmansk and Arkhangelsk oblast were published later [62]), on the content of POPs and metals in maternal blood, breastmilk and cord blood taken in Salekhard (Yamal-Nenets okrug), Norilsk and Dudinka (Taimyr okrug) [74,75] and of the POPs in the maternal blood from Arkhangelsk [69]. The preliminary results of these studies were included by fragments in the AMAP-1998 report [13], mainly with reference to “personal communication”. The AMAP-2002 report [14] contained the already-published materials of the above-mentioned Russian–Norwegian studies on the biomonitoring of contaminants in human biological media in the enumerated regions of the Russian Arctic. 

## 6. GEF/AMAP/RAIPON Project “Persistent Toxic Substances, Food Security and Indigenous Peoples of the Russian North”

In 2001, Russia’s first large-scale GEF/AMAP/RAIPON project “Persistent Toxic Substances, Food Security and Indigenous Peoples of the Russian North” was launched with the participation of the Russian Polar Fund, the Russian Association of Indigenous Peoples of the North, Siberia and the Far East (RAIPON), the Russian Ministry of Natural Resources, the Ministry of Health and Social Affairs (Northwest Public Health Research Center—NWPHRC), the Federal Service for Hydrometeorology and Environmental Monitoring, Roshydromet (RCMA and SPA Typhoon) and multiple Russian Arctic regional organizations of various departmental affiliations, as well as foreign colleagues from Norway and Canada.

The GEF/AMAP/RAIPON project of 2001–2004 initially covered four regions of the Russian Arctic: Murmansk Oblast, Nenets, Taimyr and Chukotka autonomous okrugs. Within the framework of the project, the sampling and analysis of hundreds of various biota samples (terrestrial, freshwater and marine) was carried out by the RCMA and SPA Typhoon, and biota samples were partially taken by specialists from the NWPHRC. The sampling of human biological media (blood, cord blood, breastmilk) was the full prerogative of the NWPHRC. Subsequent analysis of human samples was carried out in SPA Typhoon, and partly at the RCMA, Unilab Analyze AS (Tromsø, Norway) and the Center de Toxicologue du Quebec (Quebec, QC, Canada). All the collected samples were analyzed for three metals (Hg, Pb and Cd) and the wide list of legacy POPs.

For the first time during the expeditions of 2001–2003 (headed by Alexey Dudarev from the NWPHRC) to several coastal and mainland settlements in Chukotka autonomous okrug, hundreds of samples of human biological media of indigenous people (Chukchi and Eskimos) were collected. It turned out that, right in the coastal Chukotka (settlements of Uelen, Lorino and Lavrentiya), the highest levels of exposure of indigenous people to legacy POPs were observed (among the studied Russian Arctic regions). This phenomenon in coastal Chukotka was related to the ancient indigenous tradition of marine mammal hunting, and therefore to the regular consumption by the local people of marine mammal blubber highly contaminated by lipophilic POPs. The coastal Chukotka POP exposure levels were comparable to the highest circumpolar levels inherent to the natives of northern Canada, Greenland and the Faroe Islands.

The first results of the GEF/AMAP/RAIPON project were firstly presented at the International Congress of Circumpolar Health in 2003 in Nuuk, Greenland [63]. Some data on the exposure to POPs of coastal Chukchi and Eskimos of Uelen village [80] and data on PCB and PCDD/Fs levels in marine mammals of coastal Chukotka [49] were published before the release of the AMAP-2004 report [29]. The materials of the report published at the end of 2004 were later included in the First Regional Report of 2008 on the implementation of the “Global Monitoring Plan for POPs under the Stockholm Convention” [64] in the AMAP Report of 2009 [15] and were discussed in multiple publications in Russian and international journals [50,65,81,82,92,93,94,95].

Since 2002, Alexey Dudarev, being included in the AMAP Human Health Assessment Group as the key national Russian expert, has been a lead coauthor of a number of chapters of the three AMAP Reports “Human Health in the Arctic” of 2009, 2015 and 2021 [15,16,17]. The AMAP-2009 report [15] contained the first detailed comparison of biomonitoring data on contaminants in biota and human tissues in all circumpolar countries, including five subjects of the Russian Federation. The fifth subject (added to Murmansk Oblast, Nenets, Taimyr and Chukotka autonomous okrugs) was Kamchatka and the Commander Islands, where, in 2003–2004, within the framework of the Russian–US cooperation between the NWPHRC and the Aleutian International Association, the project “PTS, food safety and the indigenous peoples of Kamchatka and the Commander Islands” was implemented. Materials on the biomonitoring of POPs in human blood serum in five subjects of the Russian Arctic were also analyzed and compared in a joint article with Norwegian and Canadian colleagues in 2009 [65]. 

The first pilot follow-up personalized biomonitoring study of the content of legacy POPs and metals in the blood of indigenous people of coastal Chukotka (Lorino and Lavrentiya settlements) was carried out in 2007 (5 years after the first survey in 2001–2002), when the same mothers and their grown-up 5-year-old children were reexamined for comparison “puerperal → mother” and “fetus (cord blood) → child”. Maternal blood levels of POPs during the 5-year period decreased significantly (by 33–74%); blood levels of Pb decreased by 21%, while the Hg levels remained the same. The infant blood serum levels of most POPs during the five-year period increased considerably; the blood Pb levels did not change, while the Hg levels decreased by 31%. Initially lower (compared to maternal) levels of contaminants in cord blood (due to the placenta barrier) significantly increased during the 5 years in the blood of children (due to breastfeeding and further consumption of local food), reaching the initial maternal levels [82].

The AMAP-2015 report [16] contained the latest Chukotka published data, as well as the results of the Norwegian–Russian biomonitoring in the Komi Republic (Izhma and Usinsk settlements) and the Nenets Autonomous Okrug (Nelmin-Nos settlement), where the concentrations of POPs in the blood of residents of a Nenets village, first measured in 2001–2003, were compared with the levels of POPs identified in 2009–2010 (after 7–9 years): an almost twofold decrease in DDE plasma concentrations and a significant decrease levels of HCB in both women and men was found; however, there were no clear trends in PCB levels [72].

One more unique pilot study, the results of which have not been included in the AMAP-2015 report, should be mentioned. Exposure to perfluoroalkyl substances (PFASs) experienced (blood sampling of 2001–2002) by delivering women and their newborns was assessed in the industrial city of Norilsk (Taimyr Peninsula) compared to the rural towns of Urgench and Khazarasp (Aral Sea region of Uzbekistan) [78]. The observed Norilsk PFOS plasma concentrations were comparable to those in samples collected in 2001–2002 from indigenous delivering women from remote rural areas of Taimyr and Naryan-Mar (Nenets okrug) [71]; similar levels were also observed for Inuit adults of Nunavik in the Canadian Arctic. It should be noted that these two studies of PFASs were the only ones in the Russian Arctic biomonitoring history.

## 7. Recent Biomonitoring Studies in the Russian Arctic

The latest AMAP-2021 report [17] has become “loaded” with the new Russian biomonitoring data on POPs and metals in both biota and human blood from three regions of the Russian Arctic: Murmansk Oblast, Chukotka okrug and Nenets okrug. 

The three lateral EU project Kolarctic KO467 “Food and health safety in the border regions of Norway, Finland and Russia: linking industry, communities and socio-economic consequences” was implemented in 2013–2016. The Russian part of the project was carried out by the NWPHRC (headed by Alexey Dudarev) in the Pechenga district of the Murmansk Oblast, namely in the Nickel and Zapolyarny towns located close to the Ni-Cu smelter of the Pechenganickel enterprise. Practically all kinds of local biota were sampled in 2013 (fish, poultry, game, mushrooms, wild and garden berries and garden vegetables). Blood sampling involved the participation of 50 adult men and women in 2013 and 50 pregnant women in 2013–2014. A broad suite of legacy POPs and 13 metals (Pb, As, Cd, Hg, Cu, Zn, Ni, Cr, Fe, Mn, Co, Sr and V) were analyzed in the collected samples in the laboratory of the NW branch of SPA Typhoon (former RCMA) in St-Petersburg. Below, we present more detailed results obtained during the project to acquaint an English-speaking reader with Russian-language publications on the project.

In the biota of the Pechenga district, ΣDDT levels were highest in king salmon (4.5 μmg/kg ww), followed by whitefish and burbot (3.7 μg/kg ww). All other species had lower levels. As for the DDT group, the concentrations were far below the Russian maximum permissible concentrations (MPC). Concentrations of DDE and DDT in moose and birds were low. Mean concentrations of HCB in all samples of game, freshwater fish and marine cod were low. This was also the situation for the PCB group, below the Russian maximum permissible concentration for fish. Concentrations of POPs were very low in all food samples measured [30].

Analysis of metals in local foods of the Pechenga district demonstrated that the mean Pb concentrations in all Russian samples of the local fauna and flora were low (<0.05 mg/kg ww). Significant excesses of the Russian maximum permissible concentrations (MPC) of some metals in many local foods were revealed (for Ni in mushrooms—up to 30 MPC). Mushroom analyses demonstrated high concentrations of toxic elements. This points out the importance of using mushrooms as indicators of metal contamination, as well as the main food contributor to human exposure. For the first time, it has been determined that highly toxic Ni, the presence of which was found in high concentrations in mushrooms, berries and vegetables, should be considered as the most important factor of dietary exposure (and health risk) of the population of the Pechenga district [31]. The levels of metals in local foods collected in the border areas of the three participating countries of the Kolarctic project (Russia, Norway and Finland) were compared in a later joint publication [32], where the Russian data were the most comprehensible and the concentrations of metals were the highest.

Another important task of the Russian part of the Kolarctic project was the analysis of drinking water in Nickel and Zapolyarny. Samples of drinking water in both towns were highly contaminated by Ni, probably due to the neighboring mining and processing of the metals. The Ni concentration in the drinking water was 2–3-fold higher than the Russian MPC [96].

Of the forty POPs analyzed in human plasma samples collected in the Pechenga district, only HCB, HCHs, DDTs and PCBs were detectable. Among the HCHs, the β-HCH dominated; α-HCH was detected in 28% of women’s blood samples; ɣ-HCH in 50% of women and in 22% of pregnant women. The presence of HCH isomers in the blood of the examined residents was obviously associated with other (than local food) sources, since local food did not contain HCH. Among the DDTs, 4.4DDE and 4.4DDT predominated. At the same time, 4.4 DDD was found in 22% of pregnant women, which indicated the probable presence of a household “fresh” source of DDT in the maternity ward of the Nickel town. The average levels of HCB, β-HCH, 4.4DDE and 4.4DDT were in the range of 0.13–1.4 µg/L of blood serum. Of the fifteen PCB congeners analyzed in the blood of the examined persons, the largest “contribution” to the total PCB was made by the following congeners: PCB-118 (17–25%), PCB-138 and PCB-153 (11–17% each), PCB-52 (10–14%) and PCB-101 (8–9%). In general, the PCB congener “profile” in the blood of the surveyed population corresponded to that in local foods. The ∑PCBs levels in the surveyed population were low: about 1 µg/L serum (120 ng/g lipids) in pregnant women and about 1.5 µg/L serum (320 ng/g lipids) in the general population. None of the examined blood serum samples (including pregnant women) exceeded the international recommended levels for ∑PCB in the blood. The levels of HCB, 4,4DDE and PCB-153 in the blood of the population of the Pechenga region were the lowest in comparison with the other Russian Arctic regions studied previously (from the Murmansk Oblast to Chukotka in 2001–2003), and among pregnant women it was similar to that in neighboring northern Norway [66].

Analysis of metals in the blood of the surveyed men, women and pregnant women in the Pechenga district demonstrated the following ranges (min–max) of metal concentrations (mcg/L whole blood)—Pb: 3.5–108; Hg: 0.3–25.2; Cd: 0.16–2.4; As: 0.41–28.7; Mn: 7.3–305.0; Ni: 1.7–95.9; Cu: 912–2800; Zn: 3830–10,600. The highest concentrations of Mn, Co, Ni, Cu, Zn, As and Pb were observed among women, while the highest observed concentrations of Cd and Hg were among men. For the geometric mean concentrations of Hg and Pb, the male levels were up to three-fold higher compared to pregnant women. In contrast, men had lower levels of Mn, Co and As compared to women, but pregnant women still had the lowest mean concentrations. For the first time, it was reported that 50% of the surveyed men and women, and 100% of pregnant women, in the Pechenga district demonstrated a significant excess of the WHO “reference levels” of Ni in the blood (5 mcg/L). When compared with the data obtained in 2001–2003 for other Russian Arctic territories, it should be noted that the population of the Pechenga district is characterized by the lowest levels of exposure to Pb, and average levels of exposure to Hg and Cd [67].

In 2021, a joint article by members of the AMAP Human Health Assessment Group was published, summarizing the results of the MercuNorth project on the biomonitoring of Hg in the blood of pregnant women in the circumpolar Arctic (samples collected in 2010–2016). In the article, Russian data on the Pechenga district of the Murmansk oblast were used, along with materials from all eight Arctic countries that participated in the project [68].

A community-based dietary and lifestyle survey, with sampling of local terrestrial, freshwater and marine biota for analysis of environmental POPs and metals, was undertaken in spring 2016 in the Providensky district of coastal eastern Chukotka (settlements of Enmelen, Nunligran and Sireniki). This study, headed by Alexey Dudarev from the NWPHRC, was a part of the US–Russian project “Food Security and Lactic Bacteria Use in Alaska and the Bering Strait Region” (2016–2019), administered by the Institute of Northern Engineering, University of Alaska Fairbanks. Four articles in a special issue of the *IJERPH* were published in 2019, presenting the results of the study [55,56,97,98].

Concentrations of POPs in the Providensky district were very low in fish, land and marine mammal meat. However, none of the POPs analyzed exceeded the Russian MPC. 

Temporal changes in the levels of POPs in local foods in the Providensky district indicated that there was a tendency for rising levels of HCB in marine mammals. These observations were consistent with the global decline in the levels of the majority of POPs [55]. 

Analysis of additional sources of in-home food contamination (home-brewed alcohol, domestic insecticides and wash-outs from the kitchen walls) was performed in the Providensky district. The results showed high levels of HCHs, DDTs and PCBs, still representing a share of the dietary exposure of local people to POPs [55]. 

An ongoing study in the communities of the Chukchi Peninsula was the first to report contamination levels of wild plants and seafood (seaweed, blue mussels and ascidians) by 18 metals in the coastal region of the northern Bering Sea [56]. The consumption of marine mammal blubber was shown to be the main exposure route for POPs, and dietary exposure to metals was primarily from fish, seafood, wild plants, mammal meat and fowl. An algorithm for calculating the Recommended Food Daily Intake Limits (RFDILs) for certain types of local foods was developed and applied for the practical recommendations for local Chukotka natives, based on Russian and foreign standards (ADIs and TDIs oral) for POPs and metals. The developed methodology for calculating RFDILs provides the ability to take into account the simultaneous presence of several contaminants in several food products [98].

With regard to the human biomonitoring of contaminants in the body of indigenous peoples of Chukotka, a Spanish–Russian–Norwegian study showed a significant decrease (by 1.5–3 times) in the concentrations of most POPs in the blood of pregnant women in coastal Chukotka (sampling in 2014–2015) compared with the results obtained in the same region of Chukotka in 2001–2002 [84].

In 2017, the Arctic Biomonitoring Laboratory was established on the basis of NArFU (Northern (Arctic) Federal University, named after M.V. Lomonosov) in Arkhangelsk. The newly formed laboratory focused its efforts on the Nenets autonomous okrug (NAO), adjacent to the Arkhangelsk region, where, in 2017–2018, Russian–Norwegian research was conducted as a sequel of the initial biomonitoring studies of the contaminants in the biota and human tissues of local people, carried out during the implementation of the GEF/AMAP/RAIPON project in 2001–2002. 

The main POPs in fish from the area were DDTs and PCBs. However, no health risks were identified, and most of the substances showed a decreasing trend. Hg concentrations were low and were comparable to other Arctic studies. Northern pike was the only species for which Hg bioaccumulated. Cd and Pb were present in lower concentrations compared to Hg [38].

Human blood sampling in NAO was carried out in 2018 with the participation of 204 women from seven rural settlements, and the collected samples were investigated for PCBs and 17 organochlorine pesticides in blood serum [73]. Concentrations of all POPs in human serum were low, and most POPs decreased in the last 15 years (compared to that of 2001–2002). The results indicated a clear downward trend in the serum concentrations of p,p-DDE, HCB, PCB138 and PCB153 from 2002 to 2009 [72] and further to 2018. Interestingly, the most pronounced decline was observed between 2009 and 2018 [73]. It can be stated that, in the European part of the Russian Arctic, the 15-year POP temporal trends became available for the Nelmin-Nos settlement in the NAO.

A total of 297 whole-blood samples collected in 2018 in seven rural settlements of NAO were analyzed for As, Cd, Hg, Pb, Mn, Co, C and, Zn, which were generally low. The highest concentrations were found in the coastal populations, except for Co, Cd and Se, which were higher in the inland population. Pb concentrations were, however, at exceeding levels, with increased risk of nephrotoxicity [99].

## 8. Russian Regional “Hotbeds of Science” Not Involved in the AMAP Activities

When speaking about biomonitoring in the Russian Arctic during last decade, it is necessary to mention some regional “hotbeds of science” which are not involved in the AMAP activities or other international collaboration, and which are lacking unified international approaches and protocols. 

One of these scientific centers is located in the Yamalo-Nenets autonomous okrug (YaNAO). The “Scientific Research Centre of the Arctic” (SRCA) was established in 2010 in the Salekhard city of the YaNAO to conduct a wide range of research. The opening in 2011 of a branch of the SRCA in the Nadym settlement of the YaNAO allowed for keeping experienced specialists from the Research Institute of Medical Problems of the Far North of the Russian Academy of Medical Sciences, which was liquidated in Nadym two years earlier. Currently, the SRCA has seven research sectors, including environmental protection, ecological and biological research. The latter was headed during last decade by Dr. Elena Agbalyan, chief researcher. 

More than 130 samples of the YaNAO local fish (muscles and liver of muksun, nelma, broad whitefish and humpback whitefish) and 40 samples of reindeer meat (place and date of sampling not specified in the publication) were examined for contents of Cd, Pb and Ni by the atomic absorption method in an acetylene–air flame using spectrophotometer Spectr AA-50B (Varian, Sydney, Australia) at the Research Institute of Medical Problems of the Far North (Nadym, YaNAO). The highest levels of Cd (0.078 mg/kg ww) were detected in nelma liver, the highest levels of Pb (0.121 mg/kg ww) were detected in muksun liver and the highest levels of Ni (0.254 mg/kg ww) were detected in venison [40]. We also disposed some scattered data on the concentrations of Hg in the YaNAO local fish and reindeer meat; however, these materials are unpublished, and again, the place and date of sampling are not specified.

In 2016, 151 blood samples from the indigenous and nonindigenous residents of the YaNAO were collected in the Tazovsky settlement (and Tazovsky tundra), Gyd settlement (and Gydan tundra), Antipayut settlement (and Antipayutinskaya tundra), Nakhodka settlement (and Nakhodka tundra), Kutopyugan settlement (and Kutopyugan tundra) and Kharsaim settlement. A majority of the collected samples were from the Tazovsky district (89 samples) of the YaNAO. Chemical analysis of Hg in the blood samples was carried out in the Laboratory for Biotic Medicine (Moscow, Russia) using inductively coupled plasma mass spectrometry (ICP-MS) on a Nexion 300D quadrupole mass spectrometer (Perkin Elmer, Waltham, MA, USA). High levels of Hg were revealed in a significant portion of the samples taken. In particular, in the villages of Kharsaim and Kutopyugan, the portion of residents with an excess of blood Hg levels over 15 mcg/L were 37% (mean value 24 mcg/L) and 30% (mean value 26 mcg/L), respectively. The average levels of Hg in the blood from indigenous men (11.2 mcg/L) was almost five times higher than in nonindigenous men (2,3 mcg/L); in indigenous women (9.6 mcg/L), the difference was almost three times in comparison with the nonindigenous women (3.8 mcg/L) [76]. The revealed high Hg levels in the blood of the YaNAO indigenous people are extraordinary because, in the whole Russian Arctic (from the Kola Peninsula to Chukotka), the Hg levels over 20 mcg/L have been detected only sporadically.

Additionally, for the Hg analysis, the same 151 blood samples from the YaNAO residents were also analyzed (in the same laboratory using the same mass spectrometer) for the content of metals (Pb, Cd, As, Ni, Cu, Mg, Ca, Fe, Mn, Zn, Cr and Co). The average blood levels of toxic metals were relatively low: Pb—24 mcg/L; As—1.6 mcg/L; Cd—0.9 mcg/L; Ni—3.0 mcg/L. It should be noted that the latter results were published in conference proceedings, not in a peer-reviewed journal [77].

We have identified several publications on the contamination of some biota species sampled in the southern and central regions of the Karelia republic (below 64°N), which cannot be attributed to the Arctic regions. We found a small fragment from the aquatic ecology research, namely the analysis of Hg in perch sampled from four small lakes in the northern Karelian Loukhsky district (the White Sea lowland) by a team of specialists from two institutions of the Russian Academy of Sciences—Institute of Biology of the Karelian Research Center (Petrozavodsk, Karelia Republic) and the Papanin Institute for Biology of Inland Waters (Borok, Yaroslavl Oblast), which were carrying out a “long-term monitoring” (obviously since the early 2000s) of Hg in freshwater fish. Hg concentrations in perch muscles, determined by the atomic absorption spectroscopy at the Papanin Institute, were the following: in the lake Krivoye, 0.09 mg/kg ww; in the lake Sredneye, 0.14 mg/kg ww; in the lake Krugloye, 0.15 mg/kg ww; in the lake Zhemchuzhnoe, 0.37 mg/kg ww. It was concluded that the content of Hg in the muscles of perch from these small lakes was “relatively stable over the past 10–15 years” [35].

Biota and human blood concentrations of metals have also been studied in the Yakutia (Sakha) Republic. The assessment of metal levels in freshwater fish from the northern Yakutia began in the 1990s. Ajan Nyukkanov from the Yakut State Agricultural Academy (Yakutsk city) defended his candidate dissertation, “The content of heavy metals in freshwater fish of Yakutia”, in 1996, and doctoral dissertation, “Impact of natural ecotoxicants on hydrobionts in the Sakha (Yakutia)”, in 2004 (in the same year he published a monograph of the same name [44]). Unfortunately, we could not find any of Nyukkanov’s informative articles related to the biomonitoring term in any domestic or foreign scientific journals. Nyukkanov’s monograph and the dissertation contain the data on the levels of Hg, Pb and Cd in samples of muscles, liver, intestine, bones and gills from perch, crucian carp, roach and chukuchan collected in the Vilyuisky and Momsky districts of northern Yakutia. However, the given tables do not indicate the number of samples analyzed, or the specific location or the calendar year of fish sampling.

In 1972, Matvey Tyaptirgyanov started working in the laboratory of ichthyology at the Institute of Biology of the Yakutian Department of the Siberian Branch of the USSR Academy of Sciences (SBAS); in 1986, he became the head of the laboratory of aquatic ecosystems. In 1988–1991, he was the head of the ichthyology sector at the newly created Institute of Ecological Problems of the North of the Academy of Sciences of the Republic of Sakha (Yakutia). In 1994–2010, he worked as the director of the Department of Biological Resources of the Ministry of Nature Protection of the Republic of Sakha (Yakutia), managing and implementing a number of scientific projects. In 2017, he defended his doctoral thesis, “Fish of freshwater reservoirs of Yakutia (systematics, ecology, impact of anthropogenic factors)”. Being the docent of the North-Eastern Federal University named after Ammosov (Yakutsk city) before defending the dissertation, from 2014 to 2015, Matvey Tyaptirgyanov published three separate articles in the *Yakut Medical Journal* (in Russian) containing the data on Cd [45], Pb [46] and Hg [47] in fish samples. These articles were aimed at summarizing the results of multiyear expedition studies carried out in several Yakutian rivers. Again, we face the same problem: the given tables do not contain information on sampling locations (only the names of rivers without the indication of upper, middle or lower courses), on the numbers of samples analyzed and on the calendar year of fish sampling. The dissertation of Matvey Tyaptirgyanov helped us to find out that these articles combine the results of 2006–2010 studies (and earlier period) of the lower reaches of the rivers Khroma, Indigirka and Kolyma, which flow into the Arctic Ocean, and also the Vilyui and Amga rivers, which lapse into the Lena River at the latitudes of 64° N and 62° N, respectively. 

Chemical analysis of metals (Cd, Pb, Cd) was carried out in the sanitary–hygienic laboratory of the Center for Hygiene and Epidemiology in the Republic of Sakha (Yakutia) (Yakutsk city) using the atomic absorption spectrophotometer (AAS-3, Perkin-Elmer) in an air–propane flame. Similar to Nyukkanov’s results, the Tyaptirgyanov data is presented for muscles, liver, intestine, bones and gills sampled from perch, crucian carp, roach, chukuchan and also from pike, dace and broad whitefish. The authors concluded that the exceedances of Cd concentrations in Yakutian freshwater fish constitute 1–2 Russian maximum permissible concentrations (MPC), more often in polluted basins, localized in fish gills and liver [45]. Studies indicated insignificant levels of Pb in the tissues and organs of herbivorous and carnivorous fish, and few exceedances of Pb over MPC were detected in the regions of industrial mining [46]. The data obtained on Hg show 1–3 MPC exceedances in predatory fish (pike and perch) and in nonpredatory (crucian carp, roach and dace) fish in the industrial areas of diamond and gold mining [47]. It should be noted that all studies of metals in Yakutian fish were carried out by biologists–ichthyologists, who set the main goal of their research for studying the impact of contaminants on the fish population and health in conditions of their habitat pollution, but not for performing the biomonitoring of metals.

In 2017, the M.K. Ammosov North-Eastern Federal University and the Institute for Humanities Research and Indigenous Studies of the North (Yakutsk city, Russia), together with the Institute of Physiology and Basic Medicine (Novosibirsk city, Russia), carried out a human biomonitoring study in northern Yakytia. The study included 107 indigenous people (35 men and 72 women) belonging to the ethnic group of Dolgans living in the village of Yuryung-Khaya (Anabar district, Yakutia) situated on the shore of the Anabar river, 100 km from the Arctic Ocean coast. Blood serum concentrations of 13 elements (P, Sc, Ti, Cr, Mn, Fe, Ni, Cu, Zn, Rb, Sr, Cs and Pb) were analyzed by the inductively coupled plasma mass spectrometry (MS-ICP) using the Elan 9000 (Perkin Elmer, USA) at the Institute of Tectonics and Geophysics (Khabarovsk). The median levels of metals in the blood serum of the Dolgans were the following: Pb—9.5 μg/L (12.8 μg/L in men and 8.6 μg/L in women); Cr—277 μg/L; Mn—133 μg/L; Ni—57 μg/L. The authors concluded that their study revealed enhanced levels of several elements in blood serum which may affect the development of cardiovascular and other diseases among Dolgans under the conditions of the industrialization of the Arctic and expressed a hope that their study was the starting point in “the study of the microelement composition of the blood of the indigenous peoples of the Russian North” [79].

It should be noted that the above study is among several studies of the microelement status (mainly for clinical patients) which use the blood serum as a human biological medium. This phenomenon contradicts the long-established worldwide practice of studying toxic and essential elements in whole blood (which has been practiced by the AMAP for 30 years) and hampers the correct comparisons of the results of various biomonitoring studies at the circumpolar and global scales.

The Research Institute of Therapy and Preventive Medicine and the Institute of Chemical Kinetics and Combustion named after V.V. Voievodsky of the Siberian Branch of the Russian Academy of Sciences (both from Novosibirsk) carried out a study of the “elemental composition” of the whole blood of indigenous people of the coastal and inland Chukotka (the results were published in 2015, but it was not specified when the blood sampling was performed). A total of 98 blood samples of inland Chukchy from the Kanchalan village (Anadyr district, inland Chukotka) and 36 blood samples of coastal Eskimo from the Novoye Chaplino village (Providensky district, coastal Chukotka) were analyzed for 14 elements (Pb, K, Ca, Fe, Ni, Cu, Zn, Ga, Ge, Se, Br, Rb, Sr and Zr) by X-ray fluorescence analysis using synchrotron radiation (XRF SR) at the elemental analysis station of the Siberian Center for Synchrotron and Terahertz Radiation of the Institute of Nuclear Physics named after G.I. Budker of the Siberian Branch of the Russian Academy of Sciences [83]. The results presented in the article raise very serious doubts about the correctness of the performed analysis. For example, the geometric mean values of the Pb blood concentrations were 270 mcg/L in Chukchi men, 250 mcg/L in Chukchi women and 300 mcg/L in Eskimo men and women. Based on multiyear biomonitoring studies in the circumpolar Arctic (including coastal and inland Chukotka), it should be stated that the Pb concentrations presented by the authors of the above study are the highest ever found, and the average levels should be 10 times lower! In such a situation, it is unacceptable to take seriously all the work done by the authors.

The last in our list of the Russian regional “independent hotbeds of science” which are not involved in the AMAP activities, and therefore are lacking unified international approaches and protocols, but which are trying to deal with the biomonitoring of contaminants in marine biota (mainly in the water areas of the Sea of Okhotsk, the Sea of Japan and the northwestern part of the Pacific ocean), is the Vladivostok “alliance” of the Far Eastern Federal University (FEFU) and the Pacific Research Fisheries Center (TINRO-Center); the third Vladivostok’s institute (V.I. Il’ichev Pacific Oceanological Institute of the Far Eastern Branch of the Russian Academy of Sciences, POI FEB RAS) seems to work separately. These institutions during last decade have produced a dozen articles (in Russian and international journals) containing the results of biomonitoring studies of POPs and metals in marine mammals from the Chukotka coastal waters of the Bering Sea. It turned out that all these articles are based on the results of two expeditions in the summers of 2010 and 2011 to one single place—Mechigmensky Bay of the Bering Sea (Lorino village, coastal eastern Chukotka). The first article [51] presented the data on the chemical analysis of HCHs, DDTs and four metals (Pb, Cd, Hg, As) in the muscles and liver of seven gray whales (four males and three females) hunted in the summer of 2010. The second article [52] showed the results of the analysis of the same contaminants (except As) in the muscles and liver of eight walruses (five males and three females) hunted in the summer of 2011. The third [53], and several subsequent articles, contains the combined POPs results of two previous publications, i.e., data on HCHs and DDTs in the muscles and livers of gray whales and Pacific walruses. 

In the above studies, the chemical analysis of α-, β- and γ-HCH, DDT and DDE was performed using the gas chromatograph Shimadzu with an ECD (electron capture detector). The authors demonstrate unusually high levels of HCHs and DDTs in whale and walrus muscles and liver (blubber was not analyzed; unknown why), with the results presented on the lipid weight basis. It is unclear why, but the authors display the “total OCP concentration” (∑HCHs + ∑DDTs) in marine mammal tissues, apparently forgetting that the global OCPs are numerous and are not limited to HCHs and DDTs. The author’s “total OCP concentration” in the whale liver was up to 13,808 ng/g lipids, and in the walrus liver it was up to 90,263 ng/g lipids. These enormous concentrations (90,000 ng/g lipids = 90 mcg/g lipids) do not inspire confidence. The levels of ∑HCHs and ∑DDTs up to 100 mcg per kilogram wet weight each (200 mcg/kg ww together) have been detected in the blubber of whales and walruses sampled in the coastal Chukotka in 2016 [55] and in the early 2000s [29]. For the blubber tissue (up to 95% of fat, unlike 2–7% of fat in liver tissue), the wet weight is almost the same as the lipid weight, meaning that the author’s levels of POPs in the liver are 500 times higher compared to that in the blubber of Chukotka marine mammals! It just cannot be.

Chemical analysis of metals (Pb, Cd, Hg and As) in the gray whale muscles and liver [51], and in the Pacific walrus muscles and liver [52], was carried by atomic absorption spectrophotometry using the Shimadzu AA 6800. The same equipment and method was applied for analysis of Pb and Cd in the organs (kidneys, liver, spleen, intestine, testicles, lungs, heart and bones) sampled from 22 walruses (11 males and 11 females) from the same place (Mechigmensky Bay of the Bering Sea) in the summer of 2011 [54]. All metal concentrations in these three publications are presented on a dry weight basis; in this review, we will not consider these results as unacceptable. The biomonitoring of metals in the biota in the circumpolar Arctic, for many years, has used the calculations of metal concentrations on a wet weight basis, not on a dry weight basis, as was done by the authors of these articles. No unified correction factors exist; the value of the metal concentrations in the same sample, calculated in different ways (for wet and dry weight), will differ by several times. 

One last thing worth mentioning regarding the Vladivostok authors is their selective approach to the comparison of their own results with the results of other researchers. Speculations and the manipulation of data are obvious in this regard; it is not clear which contaminants and in which tissues are compared. The concentrations of contaminants in the tissues of walruses and whales of the Bering Sea, obtained in the above studies, were compared by the authors with the levels of contaminants in the tissues of whales and dolphins, reported from other regions of the planet: metals data are from the Gulf of Genoa, Mediterranean Sea, Argentine and Brazil, and POPs data are from Korean waters, Australian waters, the Black Sea, California waters, the Sea of Japan, but not from the Arctic. There is a strong belief that the authors from Vladivostok are living in “Another World” (perhaps the Eastern world) and simply ignore the abundance of results reported from different regions of the circumpolar Arctic, where, for at least 30 years, POPs and metals in different biota species have been analyzed on a wet weight basis (not on dry weight) in raw tissues (not in lipids). This contradiction excludes the possibility of comparing Vladivostok’s results with the international Arctic data obtained in the frame of circumpolar activities.

Thus, the Russian regional “hotbeds of science”, which are not involved in the AMAP activities or other international collaboration, could be characterized by the lack of unified international approaches and protocols, and therefore by the incomparability and frequent incorrectness of the results obtained.

## 9. Summarizing the Results of Russian Arctic Biomonitoring

Completing the description of the accumulated 40-year results of the biomonitoring of contaminants in the Russian Arctic, we will try to summarize the work done (Table 1 and Table 2), outlining the main achievements, gaps, problems and perspectives. The main characteristics of the Russian Arctic biomonitoring are the following:

- Relatively small number of studies carried out generally;
- Small number of studies carried out in each region;
- Regarding the biota contamination, there is no data from Komi, Turukhansky and Evenkiysky districts, and few results from Karelia, Arkhangelsk Oblast, Yamal, Taimyr and Yakutia;
- Regarding the human biomonitoring, there is no data from Karelia, Turukhansky and Evenkiysky districts, and few results from Arkhangelsk Oblast, Komi, Yamal and Yakutia;
- Most of the studies were carried out in the 1990s, followed by the large-scale GEF/AMAP/RAIPON project of 2001–2004, which covered four regions (Murmansk oblast, Nenets okrug, Taimyr and Chukotka); after that, only single projects have been implemented in a few regions;

Thus, only the Nenets okrug and the Chukotka okrug (and hardly the Murmansk Oblast) can be attributed to the regions where the biomonitoring of contaminants was carried out during last decades on several occasions, and for which the 12–15-year dynamic trends (at least “at three points”) of the content of POPs and metals in the biota and human organism were obtained. For the rest of the Russian Arctic territories, only fragmentary “cross-sections” of biomonitoring data is available, mainly obtained in the 1990s or early 2000s, which do not allow to judge either the dynamics of the processes or the current state of affairs.

Another important issue is that the majority of the studies in the Russian Arctic (more than 90%) were carried out within the framework of international projects, i.e., with cofinancing, assistance and contribution (including laboratory analyses) from foreign colleagues and partners.

In addition, it should be noted that, with the exception of a single pilot personalized cohort study in coastal Chukotka in 2007 [82], studies similar to those permanently conducted, for example, in Norway, Canada or the Faroe Islands (when the same individuals are examined for decades as they grow older, and hundreds or even thousands of people are involved in the studies), have never been performed in the Russian Arctic. Moreover, most biomonitoring studies in the Russian Arctic are characterized by a small number of observations (samples analyzed), which does not allow drawing statistically valid conclusions, comparisons or generalizations.

As for the spectrum of the analyzed POPs, in contrast to the modern circumpolar Arctic, where, in parallel with the “old” (“legacy”) POPs, the “new” (“emerging”) contaminants (polybrominated, polyfluorinated, etc.) are on the agenda of biomonitoring studies, in the Russian Arctic, only “legacy” POPs are the focus of research, as a rule. The reason for that is the limited chemical–analytical potential in the Russian Arctic and generally in Russia. There is a lack of modern laboratories with modern equipment and methodologies needed for the analysis of ultra-low concentrations of POPs and metals in biological tissues, as well as a lack of qualified specialists. In the entire set of the Russian Arctic regions, there is not a single laboratory accredited for the analysis of POPs in human biological media (particularly in blood) which are able to perform research at the modern international level. Under the current internal ban on the export of biological samples abroad, the implementation of biomonitoring studies in Russia, even in cooperation with foreign colleagues who dispose the necessary laboratory facilities, becomes problematic for Russian researchers. Very few Russian laboratories integrated into international scientific collaboration, which are able to provide high-quality results and to confirm their research quality by the regular participation in international interlaboratory comparisons, are located outside the Russian Arctic—in Moscow, Obninsk, St. Petersburg, Ufa and some other scientific centers. The Russian Arctic regional “hotbeds of science”, which are not involved in international collaboration, and which sometimes use very “special” laboratory equipment and apply some “exotic” laboratory methods, are doomed to incorrectness and the incomparability of the results obtained. 

Summing up all of the above, it must be recognized that the Arctic biomonitoring of contaminants in Russia has not yet been formed; by now, it is only a certain set of scattered results from different studies conducted in different years, which so far cannot be called a functioning system. The available Russian biomonitoring dataset may serve as a basis for the system formation in the future, which will require reasonable elaboration, substantial efforts and significant financing.

## 10. Perspectives of the Russian Arctic Biomonitoring

First of all, the elaboration of a concept and a strategy for the development of Russian Arctic biomonitoring is needed. It is clear that the priority is the updating of available scientific capacities, including the extension and radical modernization of existing chemical–analytical laboratories focused on the studying and monitoring of contaminants in the Arctic environment, and environmental health research centers dealing with the science-based assessment and prediction of exposure burdens and adverse health risks in Arctic populations. 

How to provide scientific capacities and how to “cover” (by the studies) the Russian Arctic territory with a coastline of 20,000 km in length? 

As we already pointed, there are only few chemical labs in Russia, primarily the “Typhoon” in Obninsk city and the northwestern branch of “Typhoon” in St-Petersburg (former RCMA), which are historically (for more than three decades) dealing with Arctic contaminants issues, which are accredited for the analysis of POPs and metals in biological tissues and which are regularly participating in the international (including AMAP) ring tests. 

There are also few research centers in Russia dealing with Arctic environmental health issues, primarily the Northwest Public Health Research Center in St-Petersburg, which, during almost three decades, has participated in international Arctic environmental health projects focusing on contaminants, and which is deeply integrated in the AMAP system.

The Arkhangelsk NArFU Arctic Biomonitoring Laboratory (ABL) was established in 2017. The ABL presents itself as a “specialized multidisciplinary laboratory (biomonitoring centre) for monitoring POPs in the Russian Arctic (“from the bottom”)”. They are planning “to accredit in the National Accreditation System the methodologies for the identification of inorganic and organic pollutants in food samples and human blood samples” and intend to develop a “methodology for conducting biological monitoring in the Russian Arctic” [100].

The ABL “within the governmental megagrant project has developed proposals to change the existing legal acts regulating biomonitoring”. They “revealed gaps in the current Russian legislation” in the sphere of the national (state) monitoring system regarding the contamination of traditional food consumed by indigenous peoples in the Arctic. The ABL staff put forward an idea to include into the list of objects for the mandatory systematic control (in the frame of sociohygienic monitoring headed by the Federal Service for Supervision of Consumer Rights Protection and Human Welfare, Rospotrebnadzor) the following item: “Chemical contamination of migratory species of birds, fish and wild animals consumed by indigenous peoples of the North, Siberia and the Far East of the Russian Federation”, anticipating that the “collection of the necessary data and information is becoming mandatory”. The ABL declares that “the state monitoring system is a very effective mechanism for a country as large as Russia”, and that “such changes will also help to harmonize monitoring activities in Russia with other Arctic States and to fill in the gaps in the Global Monitoring Reports and the Arctic Monitoring and Assessment Programme (AMAP) reports on persistent organic pollutants in traditional indigenous food” [100]. 

One clear problem is in the Russian system of sociohygienic monitoring (SHM), which, in the Arctic, is far from perfect. 

Indeed, the Rospotrebnadzor in the Arctic has to control a few chemical contaminants (including some PTS) in a certain few types of local foods produced by licensed companies for further sale in the retail network. Local (traditional) foods of individual hunting–fishing–gathering–growing, consumed by the local population, remain out of control by any regional or federal authorities. Thus, the Arctic SHM database does not contain any data on POPs and metals in traditional foods in the Russian Arctic regions, especially the monitoring data.

It is important to note that the Rospotrebnadzor branches in the regions of the Russian Arctic were reduced to a minimum during last two decades and are now experiencing an acute shortage of personnel, material resources (including the poor state of its laboratories) and financing. The SHM system in the Russian Arctic is flawed; the information on environmental factors (chemical contamination of water, air and soil) currently being sampled is characterized by a clear insufficiency both in quantitative and qualitative terms: scanty coverage of the controlled territories (small remote communities are almost out of control), irregular and infrequent data collection, limited list of analyzed contaminants, low objectivity and comparability (between regions) of the collected materials and obtained results and a general lack of valid information. Thus, it would be extremely difficult to oblige the Arctic regional Rospotrebnadzor branches to carry out the regular additional work of sampling numerous types of local foods in numerous settlements (particularly in the remote and hard-to-reach communities), and it would be impossible to perform a chemical analysis of the hypothetically collected samples on the spot.

In this situation, the question arises: how to develop biomonitoring in the Russian Arctic? Is it evident that there are no opportunities (financial–economic resources, equipment, specialists, etc.) to establish a wide net of chemical labs and research centers in each region of the “Arctic Zone of Russian Federation”? A scenario of strengthening those few scientific platforms available in the Arctic, and the active involvement of the above-mentioned Russian professional institutions, should be considered. If so, is there a need to understand how to build the national biomonitoring system, leaning on these few national scientific platforms called to cope with serious challenges and to deal with large volumes of research in numerous regions of the Russian Arctic? 

In our opinion, the only way to create the Russian Arctic biomonitoring system is to elaborate and launch the national Russian Arctic Contaminants Program (RACP), where the biomonitoring of contaminants will be an important structural element. The RACP should function on a permanent basis (for decades onward), it should be interdisciplinary and it should consist of several research and monitoring subprograms, managed by several constantly interacting expert groups:

- Environmental monitoring of contaminants (air, soil, water, bottom sediments, etc.) in three major ecosystem types: terrestrial, freshwater and marine;
- Biomonitoring of contaminants in biota (different wildlife species), primarily those consumed as traditional/local foods by indigenous peoples;
- Traditional diets and nutrition of indigenous peoples (dietary surveys) and dietary exposure assessments;
- Human biomonitoring (contaminants in human tissues);
- Human health assessments (health effects related to human exposure);
- Human health risk assessment, management and communication.

Each expert group in the first phase of their work should explore in detail all the appropriate information (spatial–temporal) accumulated to date, release a summarized comprehensive report and, after that, develop a perspective plan containing a regional-specific blueprint for the Russian Arctic.

New information for each subprogram will be collected by research expeditions in the frames of scientific projects, which could involve different specialists from different institutions, according to their plans. Funding of the projects should be provided by the research institutions themselves by obtaining various grants, including direct state financial support. 

Community-based monitoring is preferable, as it allows covering (in dynamics) all the scope of the RACP subprograms (environment, biota, human) in the selected places in parallel. In each region of the Russian Arctic, it is necessary to define the limited number of these “target communities” (“focal ecosystems”) and to determine the regional priorities for the contaminant measurements in certain wildlife species that represent important traditional/local foods which are frequently harvested by local (particularly indigenous) people. The list of such “target communities” in each region should be limited but “representative”; it must include the places of compact residences of indigenous peoples, the remote hard-to-reach settlements, the coastal villages (bordering the ocean/sea and large rivers/lakes) as well as the inland settlements. The choice should fall on “typical” locations where the inhabitants are engaged in sea hunting and fishing, reindeer herding, game hunting, freshwater fishing, gathering mushrooms, berries and wild plants, growing vegetables, etc. It is also important to include in the list the vicinities of industrial enterprises that pollute the environment (and the local food chains) by contaminants of local origin.

The RACP should be focused, first of all, on legacy POPs, new POPs, chemicals of emerging concern, toxic metals and essential elements. The health studies could be supplemented by the assessment of vitamins, hormones and other biochemical components in the human organism. The priorities for the human biomonitoring, health and risk assessments should be mother–child pairs, children and women of reproductive age (as vulnerable groups), but adult populations of both genders are very important to be included in the analysis as highly exposed groups for which health effects may become noticeable.

Monitoring plans (frequency of samplings and surveys) should take into account the time intervals required for the monitoring of different contaminants in various mediums in certain regions, but, of course, should be based on actual research opportunities. The formation of biobanks (archives of frozen biological samples stored for future analysis) is of great importance when regular financing is hardly possible.

## 11. Conclusions

Apart from the single studies of the late 1970s and 1980s in the southern part of the Barents Sea, the Russian Arctic biomonitoring of contaminants only actively started in the northwest part of the country (first of all, in Murmansk Oblast and Arkhangelsk Oblast) in the early 1990s, much later than the beginning of the global and circumpolar biomonitoring. The biomonitoring in the territory of the Russian Arctic has made a certain contribution to understanding the patterns of biota contamination and population exposure to POPs and metals in the circumpolar Arctic. However, it should be concluded that only a relatively small number of studies were carried out during the last 40 years; for several Russian Arctic regions, there is no up-to-date data, and for some regions, the data are scarce; most of the studies were carried out in the 1990s, followed by the large-scale GEF/AMAP/RAIPON project of 2001–2004 covering four regions; after that, single projects have been implemented in only a few regions. Only the Nenets okrug and the Chukotka okrug (and hardly Murmansk oblast) can be attributed to the regions where the biomonitoring of contaminants was carried out during last decades on several occasions, and for which the content of POPs and metals in biota and the human organism may be assessed in 12–15-year dynamic trends (at least “at three points”). For the rest of the Russian Arctic territories, only fragmentary “cross-sections” of biomonitoring data is available, mainly obtained in the 1990s or early 2000s, which do not allow judging either the dynamics of the processes or the current state of affairs. The overwhelming majority of the studies in the Russian Arctic (more than 90%) were carried out within the framework of international projects, i.e., with cofinancing, assistance and contribution (including laboratory analyses) from foreign colleagues and partners. The shortcomings of the Russian system of biomonitoring, including the weaknesses of the laboratory and research base, are considered. Perspectives of the Russian Arctic biomonitoring belong to the proposal of elaboration of the national Russian Arctic Contaminants Program (RACP). 

## Figures and Tables

**Table 1:** Russian Arctic biomonitoring of contaminants in BIOTA.

Region	Areas, Districts, Cities, Settlements	Sampling Year	Samples	Contaminants	Reference	Foreign Collaborator
**Murmansk Oblast**	Southern Barents Sea (northern coast of Kola Peninsula)	1977	sea fish, invertebrates	DDTs	Savinova et al., 1981 [20]	-
		1979; 1983	sea fish, seabirds	DDTs, PCBs	Savinova, 1991 [21]	-
		1977–1989	sea fish, seabirds	DDTs, PCBs	Savinova et al., 1995 [22]	Norway
		1991–1992	Seabirds	Cd, Zn, Cu, As, Se, Hg	Savinov et al., 2003 [23]	Norway
	Upper and middle course of Ponoy river (eastern part of Kola peninsula)	1991	peregrine falcon eggs	DDTs, PCBs, PCDD/Fs, HCHs, HCB, CHLs, mirex, toxaphenes, dieldrin	Henny et al., 1994 [24]	USA
	Pechenga district—Kuetsjarvi lake (near Nickel town) in the Pasvik River system	1991–1992	freshwater fish	Cd, Cu, Cr, Hg, Ni, Zn	Amundsen et al., 1997 [25]	Norway
	Seven Islands Archipelago—southern Barents Sea (northern coast of Kola Peninsula)	1992; 2002	Seabirds	DDTs, HCHs, HCB, CHLs, PCBs	Savinov et al., 2007 [26]	Norway
	Rybach’ya bank—southern Barents Sea (northern coast of Kola Peninsula)	1994	sea fish	Cd, Pb, Hg, Ni, Cu, Zn	Zauke et al., 1999 [27]	Germany
	Southern extremity of Kola Peninsula, White Sea	1992; 1998	harp seals	DDTs, HCHs, CBs, CHLs, PCBs, mirex	Muir et al., 2003 [28]	Canada, Norway
	Lovozero district	2001–2002	reindeers, hares, birds, fish, berries, mushrooms	DDTs, HCHs, HCB, CHLs, mirex, toxaphenes, PCBs, PCDD/Fs + Hg, Pb, Cd	AMAP-2004 Report [29]	Norway, Canada
	Pechenga district (Nickel and Zapolyarny cities area)	2013–2014	reindeers, hares, birds, fish, berries, mushrooms, vegetables	DDTs, HCHs, HCB, PCBs (fauna) + Pb, Hg, Cd, As, Ni, Cu, Zn, Cr, Fe, Mn, Co, Sr, V (fauna and flora)	Dudarev et al., 2015 [30]; Dudarev et al., 2015 [31]; Hansen et al., 2017 [32]	Norway, Finland
**Karelia** republic (northern districts)	Sorokskaya Guba of Onega Bay (Belomorsk district), White Sea	1993	ringed seals	DDTs, HCHs, HCB, CHLs, PCBs	Kostamo et al., 2000 [33]	Finland
	Chupa Guba of Kandalaksha Bay (Loukhsky district), White Sea	1999–2000	sea fish	DDTs, HCHs, CBs, CHLs, PCBs, mirex	Muir et al., 2003 [28]	Canada, Norway
	Northwestern Onega Bay (Kemsky district) at the junction with Kandalaksha Bay (Loukhsky district), White Sea	2001	ringed seals	DDTs, HCHs, CBs, CHLs, PCBs, mirex, PCDD/Fs, toxaphenes	Muir et al., 2003 [28]; Savinov et al., 2011 [34]	Canada, Norway
	Four small lakes (Krivoye, Sredneye, Krugloye and Zhemchuzhnoe) in the White Sea lowland (Loukhsky district)	2000s?	freshwater perch	Hg	Nemova et al., 2014 [35]	-
**Arkhangelsk Oblast** (northern districts)	Franz Josef Land	1991	seabirds	DDTs, HCHs, HCB, CHLs, PCBs	Savinova et al., 1995 [36]	Norway
	Franz-Josef Land; southern extremity of Novaya Zemlya	1991–1992	seabirds	Cd, Zn, Cu, As, Se, Hg	Savinov et al., 2003 [23]	Norway
	Outer Dvina Bay, White Sea	1998; 1999–2000	ringed seals, bearded seals; sea fish	DDTs, HCHs, CBs, CHLs, PCBs, mirex	Muir et al., 2003 [28]	Canada, Norway
**Nenets** autonomous okrug	Islands Vaygach, Dolgy, Kolguev	1991–1992	seabirds	Cd, Zn, Cu, As, Se, Hg	Savinov et al., 2003 [23]	Norway
	Southern Barents Sea (East Ice) to the north of Cape Kanin	1993	harp seal	DDTs, HCHs, HCB, CHLs, PCBs	Kleivane et al., 1997 [37]	Norway
	Southeastern Barents Sea; Yugor Peninsula; lower reaches of the Pechera River	1994–1995	birds, fish, hare, reindeer	DDTs, HCHs, CBs, CHLs, PCBs + Cd, Pb, Hg	RCMA data in AMAP-1998 Report [13]	-
	Southeastern Barents Sea (near Vaygach Island)	2002	ringed seals	DDTs, HCHs, CBs, CHLs, PCBs, PCDD/Fs, mirex, toxaphenes	Savinov et al., 2011 [34]	Canada, Norway
	Nelmin-Nos area	2001–2002	reindeers, hares, birds, fish, berries, mushrooms	DDTs, HCHs, HCB, CHLs, mirex, toxaphenes, PCBs, PCDD/Fs + Hg, Pb, Cd	AMAP-2004 Report [29]	Norway, Canada
	Krasnoe, Nelmin-Nos and Indiga villages	2017–2018	fish	Hg, Pb, Cd, As, Ni, Co, Cu, Zn	Sobolev et al., 2019 [38]	Norway
	Indiga village	2017–2018	fish	DDTs, CBs, CHLs, PCBs, mirex	Lakhmanov et al., 2020 [39]	Norway
**Komi** republic (northern districts)—NO data						
**Yamalo-Nenets** autonomous okrug	Not specified	2000s	freshwater fish	Cd, Pb, Ni	Agbalyan, 2012 [40]	-
	Southern part of Kara Sea; Yamal and Gydan peninsula; lower reaches of the Ob’ River	1994–1995	birds, fish, hare, reindeer	DDTs, HCHs, CBs, CHLs, PCBs + Cd, Pb, Hg	RCMA data in AMAP-1998 Report [13]	-
**Taimyr** Dolgano-Nenets District of the Krasnoyarsk kraj	Four lakes at southern foothills of Byrranga mountains, northern Taimyr	1993	freshwater fish	As, Hg, Pb, Cd, Ni, Cu, Se, Zn	Allen-Gil et al., 2003 [41]	USA
	Southeastern part of Kara Sea; Taimyr peninsula; lower reaches of the Yenisey River	1994–1995	birds, fish, hare, reindeer	DDTs, HCHs, CBs, CHLs, PCBs + Cd, Pb, Hg	RCMA data in AMAP-1998 Report [13]	-
	Yenisei River estuary near Dikson Island (Kara Sea)	1995	ringed seals	DDTs, HCHs, HCB, CHLs, PCBs	Nakata et al., 1998 [42]	Japan
	Yenisei River estuary near Dikson Island (Kara Sea)	2002	ringed seals	DDTs, HCHs, CBs, CHLs, PCBs, PCDD/Fs, mirex, toxaphenes	Savinov et al., 2011 [34]	Canada, Norway
	Khatanga area, Dudinka area	2001–2002	reindeers, hares, birds, fish, berries, mushrooms	DDTs, HCHs, HCB, CHLs, toxaphenes, mirex, PCBs, PCDD/Fs + Hg, Pb, Cd	AMAP-2004 Report [29]	Norway, Canada
**Turukhansky** district of the Krasnoyarsk kraj—NO data						
**Evenkiysky** district of the Krasnoyarsk kraj—NO data						
**Yakutia** (Sakha) republic (northern districts)	Lower Lena River	1992	ptarmigan, freshwater muksun	DDTs, HCHs	Rovinsky et al., 1995 [43]	-
	Southern part of Laptev Sea and East-Siberian Sea; New Siberian Islands, lower reaches of the Lena River and Indigirka River	1994–1995	birds, fish, hare, reindeer	DDTs, HCHs, CBs, CHLs, PCBs + + Cd, Pb, Hg	RCMA data in AMAP-1998 Report [13]	-
	Vilyuisky and Momsky districts of northern Yakutia	1990s–2000s (not specified)	fish	Hg, Pb, Cd	Nyukkanov, Kolesnikov, 2004 [44]	-
	Lower reaches of the rivers Khroma, Indigirka and Kolyma	2006–2010	fish	Cd, Pb, Hg	Tyaptirgyanov and Tyaptirgyanova, 2014, 2015 [45,46,47]	-
**Chukotka** autonomous okrug	Chaunsky district, coastal Chukotka	1993	seabirds	Cd, Hg, Fe, Mn, Zn, Cu	Kim et al., 1996 [48]	Japan
	Anadyrsky district (Kanchalan area); Chukotsky district (Lavrentiya, Lorino, Uelen)	2001–2002	reindeers, hares, birds, fish, berries, mushrooms, marine mammals (in coastal areas)	DDTs, HCHs, HCB, CHLs, mirex, toxaphenes, PCBs, PCDD/Fs + Hg, Pb, Cd	AMAP-2004 Report [29]	Norway, Canada
	Chukotsky district (Lavrentiya area)	2002	gray whales, walruses, ringed seals, bearded seals	PCBs, PCDD/Fs	Amirova et al., 2004 [49]	-
	Anadyrsky district (Kanchalan area); Chukotsky district (Lavrentiya, Lorino, Uelen)	2001–2003	reindeers, hares, birds, fish + marine mammals (Chukotsky district)	DDTs, HCHs, HCB, PCBs + Hg, Pb, Cd	Dudarev, 2012 [50]	Norway, Canada
	Southwestern coast of the Chukchi Sea (near Vankarem village)	2005	ringed seals	DDTs, HCHs, CBs, CHLs, PCBs, PCDD/Fs, mirex, toxaphenes	Savinov et al., 2011 [34]	Canada, Norway
	Mechigmensky Bay of the Bering Sea (Lorino village, coastal eastern Chukotka)	2010–2011	gray whales, walruses	HCHs, DDTs (*lipid weight for all tissues!*) + Pb, Cd, Hg, As (*dry weight for all tissues!*)	Tsygankov, 2012 [51]; Tsygankov et al., 2014 [52], Tsygankov et al., 2015 [53]	Doubtful results!
		2011	walruses	Pb, Cd	Trukhin et al., 2013 [54]	-
	Providensky district (Enmelen, Nunligran, and Sireniki villages)	2016	fish (marine, migratory, freshwater), meat of land mammals (reindeer, hare), meat and blubber of marine mammals (whale, walrus, seals), mushrooms, berries, wild plants, seafood (weeds, mollusks, ascidians)	DDTs, HCHs, CBs, CHLs, PCBs, mirex (fauna) +Pb, As, Cd, Hg, Cu, Zn, Ni, Cr, Al, Mn, Ba, Sr, Co, V, Be, Mo, Sn, Sb (fauna and flora)	Dudarev et al., 2019 [55]; Dudarev et al., 2019 [56];	USA

**Table 2:** Russian Arctic HUMAN biomonitoring of contaminants.

Region	Areas, Districts, Cities, Settlements	Sampling Year	Medium (Number of Samples); Indigenous or Not	Contaminants	Reference	Foreign Collaborator
**Murmansk Oblast**	Murmansk and Monchegorsk cities	1993	Breastmilk (30)—nonindigenous	DDTs, HCHs, HCB, CHLs, PCBs, PCDD/Fs	Polder et al., 1998 [57]	Norway
	Nickel and Monchegorsk cities	1993–1994	Maternal blood serum (50 + 51)—nonindigenous	Cu, Zn;	Odland et al., 1999 [58]	Norway
			Maternal and cord whole blood (50 mother–child pairs)—nonindigenous	Cd, Pb; Hg	Odland et al., 1999 [59,60]	Norway
	Lovozero village, Krasnoschelye village, Apatity city	1995	Whole blood of children (63 + 47 + 14)—indigenous and nonindigenous	Pb	Odland et al., 1999 [61]	Norway
	Murmansk city	2000	Breastmilk (14)—nonindigenous	DDTs, HCHs, HCB, CHLs, mirex, PCBs, PCDD/Fs, PBDEs	Polder et al., 2008 [62]	Norway
	Lovozero district (Lovozero and Krasnoschelye villages)	2001–2002	Blood serum and whole blood: general population (20 + 20), mother–child pairs (7)—indigenous	DDTs, HCHs, PCBs + Hg, Pb	Dudarev et al., 2004 [63]	Norway, Canada
				DDTs, HCHs, HCB, CHLs, mirex, toxaphenes, PCBs, PCDD/Fs, PBDEs + Hg, Pb, Cd	AMAP-2004 Report [29]; Holoubek et al., 2008 [64] (extended data); AMAP-2009 Report [15] (extended data)	Norway, Canada
	Lovozero and Krasnoschelye villages; Kola town (near Murmansk)	2001–2002	Blood serum: women (47 + 17 + 0), men (4 + 15 + 0)—indigenous; pregnant women (0 + 0 + 16)—nonindigenous	DDTs, HCHs, HCB, PCBs	Sandanger et al., 2009 [65]	Norway, Canada
	Pechenga district (Nickel and Zapolyarny cities)	2013–2014	Blood serum and whole blood: general population (50), pregnant women (50)—nonindigenous	DDTs, HCHs, HCB, PCBs + Hg, Pb, Cd, As, Ni, Mn, Co, Cu, Zn	Dudarev et al., 2016 [66,67]; Adlard et al., 2021 [68] (fragment on maternal Hg)	Norway, Finland
**Karelia** republic (northern districts)—NO data						
**Arkhangelsk Oblast** (northern districts)	Arkhangelsk city	1993	Maternal blood serum (50)—nonindigenous	Cu, Zn	Odland et al., 1999 [58]	Norway
			Maternal and cord whole blood (50 mother–child pairs)—nonindigenous	Cd, Pb, Hg	Odland et al., 1999 [59,60,61]	Norway
		1996	Maternal blood serum (27)—nonindigenous	DDTs, HCHs, HCB, CHLs, mirex, PCBs	Sandanger et al., 2003 [69]	Norway
	Arkhangelsk and Severodvinsk cities	1996–1997	Breastmilk (51 + 50)—nonindigenous	DDTs, HCHs, HCB, CHLs, PCBs	Polder et al., 2003 [70]	Norway
	Arkhangelsk city	2000	Breastmilk (23)—nonindigenous	DDTs, HCHs, HCB, CHLs, mirex, PCBs, PCDD/Fs, PBDEs	Polder et al., 2008 [62]	Norway
**Nenets** autonomous okrug	Naryan-Mar city	1996–1997	Breastmilk (20)—indigenous and nonindigenous	DDTs, HCHs, HCB, CHLs, PCBs	Polder et al., 2003 [70]	Norway
	Lower Pechora River area, mainly Nelmin-Nos village	2001–2002	Blood serum and whole blood: general population (32), mother–child pairs (21)—indigenous	DDTs, HCHs, PCBs + Hg, Pb	Dudarev et al., 2004 [63]	Norway, Canada
				DDTs, HCHs, HCB, CHLs, mirex, toxaphenes, PCBs, PCDD/Fs, PBDEs + Hg, Pb, Cd	AMAP-2004 Report [29]; Holoubek et al., 2008 [64] (extended data); AMAP-2009 Report [15] (extended data)	Norway, Canada
	Naryan-Mar city	2002	Maternal blood serum (12)—indigenous and nonindigenous	PBDEs, PFOS	Odland et al., 2006 [71]	Norway
	Lower Pechora River area	2001–2002	Blood serum: pregnant women (38), women (31), men (13)—indigenous	DDTs, HCHs, HCB, PCBs	Sandanger et al., 2009 [65]	Norway, Canada
	Nelmin-Nos village	2009–2010	Blood serum and whole blood: women (87), men (22)—indigenous	DDTs, HCHs, HCB, CHLs, mirex, PCBs + Hg, Pb, Cd	Rylander et al., 2011 [72]	Norway
	Seven rural coastal and inland settlements: Bugrino, Varnek, Amderma, Indiga, Shoina, Nelmin-Nos, Krasnoe	2018	Blood serum of women (204)—indigenous and nonindigenous	HCHs, DDTs, CHLs, aldrin, mirex, CBs, PCBs	Varakina et al., 2021 [73]	Norway
			Whole blood of general population (297)—indigenous and nonindigenous	As, Cd, Hg, Pb, Mn, Co, Cu, Zn	Sobolev et al., 2019 [38]	Norway
**Komi** republic (northern districts)	Izhma and Usinsk settlements	2009–2010	Blood serum and whole blood of general population: women (25 + 25), men (25 + 25)—indigenous	DDTs, HCHs, HCB, CHLs, mirex, PCBs + Hg, Pb, Cd	Rylander et al., 2011 [72]	Norway
**Yamalo-Nenets** autonomous okrug	Salekhard city	1995	Maternal and cord whole blood; breastmilk (21 mother–child pairs)—nonindigenous	Hg, Pb, Cd, Ni	Klopov, 1998 [74]	Norway
		1996–1998	Maternal blood serum; breastmilk (31)—nonindigenous	DDTs, HCHs, HCB, CHLs, PCBs	Klopov et al., 1998 [75]	Norway
	Tazovsky village (and Tazovsky tundra), Gyd village (and Gydan tundra), Antipayut village (and Antipayutinskaya tundra), Nakhodka village (and Nakhodka tundra), Kutopyugan village (and Kutopyugan tundra) and Kharsaim village	2016	Whole blood of general population (151)—indigenous	Pb, Cd, Ni, Hg; As, Cu, Mg, Ca, Fe, Mn, Zn, Cr, Co	Agbalyan, 2012 [40]; Agbalyan and Shinkaruk, 2018 [76]; Agbalyan, 2020 [77]	- -
**Taimyr** Dolgano-Nenets District of the Krasnoyarsk kraj	Norilsk city	1995	Maternal and cord whole blood; breastmilk (21 mother–child pairs)—nonindigenous	Hg, Pb, Cd, Ni	Klopov, 1998 [74]	Norway
	Norilsk and Dudinka cities	1995–1996	Maternal blood serum; breastmilk (49 + 27)—nonindigenous	DDTs, HCHs, HCB, CHLs, PCBs	Klopov et al., 1998 [75]	Norway
	Khatanga area, Dudinka area, Norilsk city	2001–2002	Blood serum and whole blood: mother–child pairs (29 + 38 + 10), general population (5 + 0 + 0)—indigenous and nonindigenous	DDTs, HCHs, PCBs + Hg, Pb	Dudarev et al., 2004 [63]	Norway, Canada
				DDTs, HCHs, HCB, CHLs, mirex, toxaphenes, PCBs, PCDD/Fs, PBDEs + Hg, Pb, Cd	AMAP-2004 Report [29]; Holoubek et al., 2008 [64] (extended data); AMAP-2009 Report [15] (extended data)	Norway, Canada
	Lower Yenisei River area; Norilsk city	2001–2002	Blood serum: pregnant women (69 + 59), women (40 + 0), men (12 + 0)—indigenous and nonindigenous	DDTs, HCHs, HCB, PCBs	Sandanger et al., 2009 [65]	Norway, Canada
	Taimyr (not specified)	2002	Maternal blood serum (12)	PBDEs, PFOS	Odland et al., 2006 [71]	Norway
	Norilsk city	2001	Maternal and cord whole blood and plasma (7 mother–child pairs)	PFASs (PFOS, PFOA, PFNA, FOSA, PFHxS, PFUnDA)	Hanssen et al., 2013 [78]	Norway
**Turukhansky** District of the Krasnoyarsk kraj—NO data						
**Evenkiysky** District of the Krasnoyarsk kraj—NO data						
**Yakutia** (Sakha) republic (northern districts)	Yuryung-Khaya village (Anabar district)	2017	Blood *serum* (35 men and 72 women = 107)—indigenous Dolgans	Pb, Ni, Cu, Zn, Cr, Mn, Fe, Sc, Ti, Rb, Sr, Cs	Sivtseva et al., 2020 [79]	-
**Chukotka** autonomous okrug	Uelen village (Chukotsky district)	2001	Blood serum (50)—indigenous	DDTs, HCHs, HCB, CHLs, mirex, toxaphenes, PCBs	Sandanger et al., 2003 [80]	Norway
	Chukotsky district, Iultinsky district, Anadyrsky district, Anadyr city	2001–2003	Blood serum and whole blood: mother–child pairs (47 + 5+39 + 12); general population (30 + 0 + 50 + 0)—indigenous and nonindigenous	DDTs, HCHs, PCBs + Hg, Pb	Dudarev et al., 2004 [63]	Norway, Canada
				DDTs, HCHs, HCB, CHLs, mirex, toxaphenes, PCBs, PCDD/Fs, PBDEs + Hg, Pb, Cd	AMAP-2004 Report [29]; Holoubek et al., 2008 [64] (extended data); AMAP-2009 Report [15] (extended data)	Norway, Canada
	Chukotsky district, Anadyrsky district, Anadyr city.	2001–2003	Breastmilk (27 + 21 + 7)—indigenous	DDTs, HCHs, HCB, CHLs, mirex, toxaphenes, PCBs, PCDD/Fs, PBDEs	AMAP-2004 Report [29]; Holoubek et al., 2008 [64]	Norway, Canada
	17 settlements in coastal Chukotsky district and inland Anadyrsky district	2001–2002	Maternal blood, cord blood and breastmilk (48 mother–child pairs)—indigenous	DDTs, HCHs, HCB, CHLs, mirex, toxaphenes, PCBs + Hg, Pb, Cd	Anda et al., 2007 [81]	Norway
	Uelen village (Chukotsky district), Kanchalan village (Anadyrsky district)	2001–2002	Blood serum: pregnant women (59 + 67), women (26 + 28), men (24 + 14)—indigenous	DDTs, HCHs, HCB, PCBs	Sandanger et al., 2009 [65]	Norway, Canada
	Lorino and Lavrentiya villages (Chukotsky district)	2007 vs. 2001–2002	Blood serum and whole blood: 17 mother–child pairs (13 + 4)—indigenous (personalized follow-up)	DDTs, HCHs, HCB, PCBs, CHLs + Hg, Pb	Dudarev et al., 2010 [82]	Norway
	Kanchalan village (Anadyr district) and Novoye Chaplino village (Providensky district)	not specified	Whole blood (98 + 36)—indigenous	Pb, Ni, Cu, Zn, Fe, K, Ca, Ga, Ge, Se, Br, Rb, Sr, Zr	Gyrgolkau et al., 2015 [83]	-
	Inland and coastal districts, incl. Chukotsky district	2014–2015	Maternal blood serum (250), incl. (63)	DDTs, HCHs, HCB, PCBs, PeCB, mirex, PBDEs	Bravo et al., 2019 [84]	Spain, Norway
